# Student athlete well-being framework: an empirical examination of elite college student athletes

**DOI:** 10.3389/fpsyg.2023.1171309

**Published:** 2023-06-15

**Authors:** Shintaro Sato, Keita Kinoshita, Midori Kondo, Yuki Yabunaka, Yaeko Yamada, Hironobu Tsuchiya

**Affiliations:** ^1^Faculty of Sport Sciences, Waseda University, Tokyo, Japan; ^2^Graduate School of Sport and Exercise Sciences, Osaka University of Health and Sport Sciences, Kumatori, Osaka, Japan; ^3^Department of Health and Sport Management, Osaka University of Health and Sport Sciences, Kumatori, Osaka, Japan

**Keywords:** well-being, student athletes, life satisfaction, happiness, organizational citizenship behavior

## Abstract

The current study proposes a multidimensional student athlete well-being framework (SAWBF). The authors used 12 items to capture SAWBF comprised of four well-being dimensions (i.e., physical, hedonic, psychological, and social well-being). To empirically assess the reliability and validity of the framework, data from elite collegiate student athletes in Japan (*N* = 546) were procured. The results indicated sufficient convergent and discriminant validities of SAWBF. The authors also assessed predictive validity correlations of the framework by focusing on the oft-supported well-being outcome–organizational citizenship behavior, which were also found to be associated with SAWBF. The findings indicated the usefulness of SAWBF; and coaches and staff members can utilize the framework to multi-dimensionally understand well-being status of their student athletes, potentially boosting adaptive behaviors.

## 1. Introduction

Many student athletes demonstrated outstanding performances in the premier international competitions such as Olympic Games (Pompliano, 2021; Saito, 2021), receiving a great deal of societal attention in various countries. Companies can also make endorsement contracts with student athletes to leverage their popularity to boost the growth of businesses (Dukurs, 2021). As such, student athletes now are not just students, but public figures to some extent. Student athletes thereby experience a variety of stressors both on and off the fields, which may affect their well-being (Rice et al., 2016; Sallen et al., 2018). Well-being among student athletes has become one of the most important concerns for student athlete organizations such as the National Collegiate Athletic Association (NCAA) in the United States and Japan Association for University Athletics and Sport (UNIVAS) in Japan. Although these upper echelon organizations have offered guidelines to protect student athletes’ well-being, they have not been as effective as expected due to the challenges regarding the conceptual operationalization and practical application.

First, one of the challenges is associated with the conceptual operationalization. Well-being is a relatively new concept interchangeably used with quality of life, life satisfaction, and/or happiness (Osman and Ismail, 2018). Although scholars have identified well-being as a multi-dimensional construct (Kern et al., 2015; Sato et al., 2022), unidimensional operationalizations are still prevalent by simply utilizing global assessments of life satisfaction (Diener et al., 2000a,b), happiness (Uusitalo-Malmivaara, 2012), and subjective well-being (Blanchard et al., 2009). Nevertheless, such conceptualizations and operationalizations are limited in their abilities to holistically capture well-being. In the current research, we proposed a four-dimensional student-athlete well-being framework (SAWBF), which focuses on physical, hedonic, psychological and social well-being aspects. Such multi-dimensional well-being perspectives can benefit student-athletes as well as practitioners (e.g., coaches, staff members) as to how student-athletes’ well-being should be protected and leveraged to enjoy fruitful adaptive outcomes.

Second, practical applications of well-being have also been considered a challenging task. Though well-being as a term has received keen attention from practitioners including coaches and staff members, whether it is deliberately measured and utilized for developing better sport environment is questionable, presumably because of two reasons: bulky measurement tools and unclear benefits of the outcomes. Scholars have utilized bulky multi-dimensional well-being scales such as 42-item Psychological Well-being Scale (Ryff, 1989) and 33-item Social Well-being Scale (Keyes, 1998). Yet, these scales impose considerable efforts to respondents. Although there are a wide variety of ways to measure well-being (Ryff, 1989; Huta, 2016; Teresi et al., 2017; Pavot, 2018), context-specific and efficient instruments that focus on student athletes have been insufficient. In addition, despite the considerable scholarly efforts that explore antecedents of student athletes’ well-being (Lundqvist and Sandin, 2014; Lundqvist and Raglin, 2015), coaching staff on sites may put more importance on outcomes rather than antecedents. Well-being is undoubtedly an important concept for student athletes, but it should be more encouraging for practitioners if scientific evidence highlights the adaptive outcomes of student athletes’ well-being. Hence, the goals of the current research are to (1) conceptualize and empirically validate the measurement tools for the multidimensional student athletes’ well-being, and (2) assess the predictive validity association with a potential well-being outcome–organizational citizenship behavior. The current research significantly contributes to the literature by providing student athlete well-being framework (SAWBF), which efficiently measures multi-dimensional well-being and highlighting a potential adaptive outcome of SAWBF–organizational citizenship behavior.

## 2. Literature review

### 2.1. SAWBF as multi-dimensional student athlete well-being

As student-athletes have been increasingly gathering attention from society, it is important to deeply understand their well-being (e.g., Reardon et al., 2019; Graupensperger et al., 2020). However, there is still a lack of universal agreement regarding the conceptualization of student-athlete well-being. Scholars more recently argue that mental well-being consists of hedonic and eudaimonic well-being (Ryan and Deci, 2001; Keyes et al., 2008; Huta and Ryan, 2010). In line with the literature, the integration of both hedonic and eudaimonic perspectives into the conceptualization of mental well-being, considering the student-athlete well-being as multi-dimensional, helps offer a comprehensive understanding of student-athlete well-being (Huta and Ryan, 2010; Huta and Waterman, 2014).

Specifically, hedonic well-being is originated from the tenet of *Hedonia*, which refers to a pursuit of personal feeling of pleasure (Ryan et al., 2008). Therefore, hedonic well-being often comprised of the presence of positive feeling, absence of negative feeling, and satisfaction with global life or a specific domain of life (Diener and Ryan, 2009). Hedonic well-being is often interchangeably used with other constructs: happiness, emotional well-being, and subjective well-being. As hedonic well-being is a significant indicator of health and performance (e.g., Lyubomirsky et al., 2005; Keyes, 2006), scholars have traditionally measured athletes’ well-being as an essential marker of an athletic success applying the hedonic perspective (*cf.*, Lundqvist, 2011). Although the context is slightly different from the current research, Kinoshita et al. (2022a,b) focused on hedonic well-being among Canadian youth athletes (measured by positive affect and life satisfaction scales) and found the association with basic psychological need satisfaction (i.e., autonomy, competence, and relatedness). Similarly, Lemelin et al. (2022) also identified athlete well-being is an integral part of student athletes, which can be enhanced by autonomy support of both parents and coaches. Accordingly, previous literature identified hedonic well-being plays an important role, the present study includes hedonic well-being as a significant component of student-athlete well-being.

In contrast, eudaimonic well-being is originated from Aristotles’ perspective also known as Nichomachean ethics (Rowe and Broadie, 2002), which argues positive human functioning. Ryff’s model of psychological well-being (Ryff, 1989) is the most referred theoretical framework to operationalize eudaimonic well-being (Huta and Waterman, 2014). Therefore, psychological well-being, defined as fulfillment and a sense of purpose or meaning: “Doing what is worth doing” (Ryan and Deci, 2001, p. 145), has been often applied to measure eudaimoic well-being. Additionally, Keyes (2006) proposed that social well-being (e.g., social integration) is also a significant component of eudaimonic well-being. According to Keyes (2006), social well-being refers to “individuals’ perceptions of the quality of their relationships with other people, their neighborhoods, and their communities” (p. 5). Keyes’ proposal is consistent with World Health Organization (2004) argument that social aspects impact individual functioning; thus, psychological well-being and social well-being has been concurrently assessed to understand eudaimonic well-being in previous empirical studies (*cf.*, Brandel et al., 2017). A recent study that focused on NCAA Division I student-athletes demonstrated that student-athletes with various engagement at schools (e.g., communicate with faculty members and other students) are more likely to report high psychological well-being (Kim et al., 2020). Moreover, Gabana et al. (2022) also put the importance on social well-being among student-athletes and studied how it can be enhanced by the gratitude intervention. As such, these empirical studies identified psychological and social well-being as integral components of well-being, the current research also included them as a subcomponent of eudaimonic well-being in student-athlete well-being.

Lastly, in addition to hedonic and eudaimonic perspectives in student-athlete well-being, physical well-being of college athletes is a significant concern. Scholars demonstrated physical illness and injury is a significant risk factor associating with mental illness and suicide mortality in athletes (Rao and Hong, 2016; Reardon et al., 2019). As a large-scale study demonstrated that college-athletes suicide mortality in the U.S. occurs with the rate of about 1/100,000 (Rao et al., 2015), well-being of the physical aspect in student-athlete should not be overlooked.

### 2.2. The outcome of student athlete well-being: Predictive validity correlations of SAWBF

Previous literature has advanced the science of well-being in various research contexts (Kinoshita et al., 2022a,b; Park et al., 2022; Sato et al., 2022). For example, Park et al. (2022) found that individuals who involve with professional sport team communities are psychologically vitalized, resulting in higher well-being. Sato et al. (2022) also found that hosting mega sporting events (i.e., Rugby World Cup 2019) played an important role in boosting residents’ well-being. Kinoshita et al. (2022a,b) focused on the relationship between sport participation levels and military officers’ well-being and found that those who have higher well-being are more likely to participate in sport with eudemonic motives (e.g., learning and skill development). These studies focusing on antecedents of well-being have contributed to the understanding of the mechanism as to how scholars and practitioners can enhance well-being in the society. Nevertheless, it is imperative to reveal the outcomes of well-being as individuals and organizations can better understand the benefits of well-being enhancement, accelerating the trends in well-being research.

Well-being has been found to be associated with various positive outcomes such as quality social relationships and work productivity (Lyubomirsky et al., 2005; Reschly et al., 2008; Seligman, 2011). One of the important outcomes that the current study particularly pays attention to is organizational citizenship behavior (OCB; Dunn et al., 2014; Kansky, 2017). OCB is defined as behavior not directly recognized by the formal reward system but that which contributes to organizational effectiveness (Smith et al., 1983). OCB has been recognized as a foremost imperative construct because it can facilitate organizational performance, productivity, and efficacy (Podsakoff et al., 2009; Koopman et al., 2016).

Scholars have considered that OCB can play a vital role in effective management of sport teams (Aoyagi et al., 2008; Love and Kim, 2019). With the empirical data derived from university student athletes in the United States, Aoyagi et al. (2008) validated multidimensional conceptualization of OCB (i.e., helping, civic virtue, and sportspersonship). Helping is characterized by athletes’ helping behavior to overcome or prevent problems; Civic virtue indicates athletes’ responsible engagement with the life of their teams; and sportspersonship is illustrated as tolerating problems and challenges with positive attitude (Aoyagi et al., 2008). These aspects of OCB are indeed beneficial to sport teams, and if the relationship between well-being and OCB is empirically validated, it will be convincing evidence for coaching staff and managers of sport organizations to invest resources to further accelerate the well-being enhancement trends.

Although research that examines the relationship between well-being and OCB among athletes has been scarce, there are several important contributions in the contexts of business management and public health. Physical well-being can be a foundation for functioning. A study conducted by Chen and Kao (2011) found that physical well-being (i.e., physical self-efficacy) is associated with OCB among Taiwanese police officers. The sample population of the current research is sufficiently similar in a sense that physical ability is also a central part of lives in student athletes. With regard to hedonic well-being, Rego et al. (2010) surveyed employees of 14 companies in Portugal and found that employees’ hedonic well-being (e.g., pleasure) is positively associated with OCB. Similarly, Xu et al. (2019) derived data from a telecommunication company in China and found that affective well-being, interchangeably used with hedonic well-being, was positively associated with OCB (Xu et al., 2019). The aforementioned study conducted by Aoyagi et al. (2008) also revealed that OCB is positively associated with athlete satisfaction (e.g., strategy), leadership (e.g., social support) and team cohesion, which are proximities of psychological and social well-being (Ryff, 1989; Huta and Waterman, 2014). Hence, it is reasonable to argue that student athletes’ well-being is associated with OCB.

## 3. Methods

### 3.1. Participants

The target population for this study was elite student athletes in Japan. We employed a convenience sampling method to procure data. Specifically, we sent a URL link and QR code generated by Qualtrics to coaching staff members (e.g., head coaches, managers) in officially approved top sport teams in Japanese universities. The coaching staff members shared online survey link and/or QR code with student athletes to ask for participation. A total of 556 student athletes from 13 Japanese universities in Kanto area (e.g., Tokyo, Kanagawa, Saitama) who compete in 23 sports (e.g., rugby, basketball, soccer, kendo) participated in the survey. After removing 10 participants who did not answer a significant number of well-being items, data from 546 student athletes were retained for further analyzes (387 males; M_age_ = 19.83 years, SD = 1.00).

### 3.2. Measures

SAWBF consists of four dimensions – physical, hedonic, psychological, and social well-being. To measure the multi-dimensional well-being scale, it is imperative to select reliable scales and items based on previous literature (e.g., Keyes et al., 2008; Diener and Ryan, 2009). We first reviewed and summarized the literature that measured constructs of our interests (Ryff, 1989; Ryff and Keyes, 1995; Diener and Ryan, 2009; Van Hoecke et al., 2014; Rena et al., 2019). However, well-being related scales in the literature are often too lengthy (e.g., Ryff, 1989; Keyes, 1998). Multi-dimensionally measuring student athletes’ well-being with a concise manner is particularly important when it comes to practical value. First, the first and second authors chose items capturing one of the well-being dimensions. Second, other co-authors discussed the list of items to further reduce and refine the items. Third, we discussed with coaches and staff members to reach the realistic number of items that are also understandable from the practical perspectives.

Physical well-being of student athletes was measured with two items, capturing self-rated health (e.g., Van Hoecke et al., 2014; Kekäläinen et al., 2017; Reysen et al., 2017). Hedonic well-being of student athletes was often operationalized with affect and life satisfaction (Diener and Ryan, 2009). In the current research, we adopted two items from previous research (e.g., Rena et al., 2019), and slightly modified them to be consistent with the student-athlete contexts. Eudaimonic well-being has two different dimensions–psychological and social well-being (Ryan and Deci, 2001; Keyes, 2006). We measured psychological well-being of student athletes with five items inspired by the past research (e.g., Ryff, 1989; Ryff and Keyes, 1995; Kouali et al., 2020). Social well-being of student athletes was measured with three items adopted from past research (e.g., Keyes, 2006). All items were slightly modified to increase face validity in the context of student athletes, and they stemmed with “*During the past month, how often did you feel about each statement*” on a 7-point scale ranging from 1 = *Never* to 7 = *every day*.

Organizational citizenship behavior measurement used in previous literature was occupation-domain oriented. We, therefore, slightly modified the items adopted from previous literature to increase the content validity in the student athlete context. Helping was measured with three items (e.g., I help other teammates if they fall behind in his sport; Podsakoff et al., 1997; Aoyagi et al., 2008). Civic virtue was evaluated based on three items (e.g., I provide constructive suggestions about how the team can improve its effectiveness; Podsakoff et al., 1997; Aoyagi et al., 2008). We utilized three reverse-coded items to measure sportspersonship (e.g., I always focus on what is wrong with our situation, rather than the positive side; Podsakoff et al., 1997; Høigaard et al., 2015). The scale has been utilized and validated in the previous literature and each item was measured on a 7-point scale, ranging from 1 = *Strongly disagree* to 7 = *Strongly agree*. The construct reliability scores for the above factors ranged from 0.79–0.92, demonstrating a sufficient internal consistency. It is also important to mention that we followed an established procedure (Yoshida et al., 2021) to back-translate all items into Japanese as our sampling frame was college student athletes in Japan. The results of descriptive statistics, correlations, and square root of average variance extracted (AVE) were presented in Table 1.

**Table 1:** Descriptive statistics and inter-item correlation.

	Constructs	Mean	SD	1	2	3	4	5	6	7
1	Physical Well-being	4.21	1.44	0.85^a^						
2	Hedonic Well-being	5.64	1.17	0.44	0.93^a^					
3	Psychological Well-being	5.26	1.05	0.50	0.70	0.75^a^				
4	Social Well-being	5.41	1.07	0.43	0.48	0.61	0.72^a^			
5	OCB–Helping	4.83	1.17	0.36	0.47	0.56	0.54	0.84^a^		
6	OCB–Civic Virtue	4.98	1.26	0.36	0.46	0.55	0.54	0.71	0.82^a^	
7	OCB–Sportspersonship	3.15	1.22	−0.10	−0.24	−0.21	−0.27	−0.12	−0.18	0.75^a^

^a^Square root of AVE; OCB, Organizational citizenship behavior.

### 3.3. Data analysis

Data analyzes were executed by using IBM SPSS 28.0 and Amos 28.0 statistics software programs. First, we assessed the discriminant and convergent validity of the measurement model by running a confirmatory factor analysis (CFA). The average variance extracted (AVE) values greater than 0.50 were set as the benchmark. We compared the square root of AVE values with the inter-correlations of each factor (Fornell and Larker, 1981) to evaluate the discriminant validity of the focal factors. For model fit indices, we employed goodness of fit index (GFI), adjusted goodness of fit index (AGFI), comparative fit index (CFI), Tucker-Lewis coefficient (TLI), root mean square error of approximation (RMSEA), and the standardized root mean square residual (SRMR) based on the previous literature (Little, 2013) with the cutoff values of 0.90 for GFI and AGFI (Hair et al., 2010), 0.95 for CFI and TLI (Hu and Bentler, 1999), and 0.08 or lower for RMSEA and SRMR (Browne and Cudeck, 1993; Hu and Bentler, 1999). Second, we further examined factorial structure of SAWBF by comparing several competing models. Specifically, (1) single factor model in which all items are loaded to one factor, (2) three factor model with physical, hedonic, and eudaimonic well-being (i.e., psychological and social well-being) factors, and (3) four factor model that consists of physical, hedonic, psychological, and social well-being factors were compared. In this analysis, we used CFI, TLI, RMSEA, SRMR, and Akaike’s information criterion (AIC) to identify the best model (Bozdogan, 1987). Lastly, following the established procedure (Anderson and Gerbing, 1988), we conducted latent variable-based Structural Equation Modeling (SEM) after the CFA to assess the predictive validity correlations of SAWBF by setting the oft-supported outcomes of well-being – organizational citizenship behavior – as the dependent variables. The associations between well-being dimensions and organizational citizenship behavior were tested by computing a 95% bias-corrected confidence interval using SPSS Amos 200 bootstrapped resamples.

## 4. Results

### 4.1. Testing measurement model

The results of confirmatory factor analysis (CFA) demonstrated that fit indices of the measurement model were also acceptable: *χ*^2^/*df* = 2.21, GFI = 0.93, AGFI = 0.91, CFI = 0.97, TLI = 0.96, SRMR = 0.04, RMSEA = 0.05 (Bollen, 1989; Kline, 2005; Little, 2013). Factor loadings for items were all greater than 0.60, and AVEs ranged from 0.52 to 0.86, confirming convergent validity of the measurement model (Fornell and Larker, 1981). Correlations among factors ranged from −0.27 to 0.71, and the square root of AVE values were greater than all inter-factor correlations, indicating that discriminant validity of measurement model was acceptable (Fornell and Larker, 1981; Table 2).

**Table 2:** Factor loadings, construct reliability, and AVE (*n* = 546).

Constructs and measurement items	*λ*	AVE	CR
Physical well-being			
I am satisfied with my physical health	0.93	0.73	0.84
I am satisfied with my body	0.77		
Hednic Well-being			
I enjoy my sport	0.90	0.86	0.92
I am happy that I am playing my sport	0.95		
Psychological Well-being			
As an athlete, I feel that I continue to learn more about myself.	0.72	0.56	0.87
My sport has a sense of direction or meaning to life	0.70		
I am confident to engage in sport	0.88		
I feel positive about myself as an athlete	0.71		
I am good at managing the responsibilities as an athlete	0.73		
Social well-being			
I have satisfying relationships with my coaches	0.72	0.52	0.76
I have satisfying relationships with my teammates	0.63		
I belong to this team	0.80		
Organizational citizenship behavior–helping			
I help my teammates if they falls behind in my team	0.87	0.70	0.87
I willingly share my expertise with other teammates	0.82		
I willingly give of my time to help teammates who have sport-related problems	0.81		
Organizational citizenship behavior–civic virtue			
I provide constructive suggestions about how the team can improve its effectiveness	0.90	0.68	0.86
I am willing to risk disapproval to express my beliefs about what’s best for the team	0.82		
I attend and actively participate in team meetings	0.74		
Organizational citizenship behavior–sportspersonship			
I always focus on what is wrong with the situation, rather than the positive side (*R*)	0.68	0.56	0.79
I consume a lot of time complaining about trivial matters (*R*)	0.84		
I always find fault with what other teammates are doing (*R*)	0.71		

*x*^2^/df = 2.21, GFI = 0.93, AGFI = 0.91, CFI = 0.97, TLI = 0.96, SRMR = 0.04, RMSEA = 0.05; (R) = Reverse-coded.

### 4.2. The factorial structure of SAWBF

SAWBF consists of four well-being factors (physical, hedonic, psychological, and social). Nevertheless, previous literature has utilized unidimensional well-being (Diener et al., 1999; Marsh et al., 2020) or combined psychological and social well-being into a single factor of eudaimonic well-being (Ryff, 1989). To ensure the factorial structure of SAWBF, therefore, we compared the four factor model with competing single and three factor models. The results showed that the four factor model demonstrated the best fitting model (*χ*^2^ = 129.38, *df* = 48, CFI = 0.98, TLI = 0.97, RMSEA = 0.056, SRMR = 0.029, and AIC = 18823.758) compared to the single factor model (*χ*^2^ = 3916.88, *df* = 198, CFI = 0.44, TLI = 0.40, RMSEA = 0.185, SRMR = 0.274, and AIC = 36990.783) and three factor model (*χ*^2^ = 270.01, *df* = 51, CFI = 0.94, TLI = 0.92, RMSEA = 0.089, SRMR = 0.045, and AIC = 18958.386). The findings indicate that the factorial structure of SAWBF deemed appropriate (Table 3).

**Table 3:** Model comparison for factorial structure assessment.

	Model	*χ*^2^	*df*	CFI	TLI	RMSEA	SRMR	AIC
1	Single factor model	3916.88	198	0.44	0.40	0.185	0.274	18958.783
2	Three factor model	270.01	51	0.94	0.92	0.089	0.045	36990.783
3	Four factor model	129.38	48	0.98	0.97	0.056	0.029	18823.758

### 4.3. The association between SAWBF and organizational citizenship behavior

To capture the overall associations between SAWBF and organizational citizenship behavior, we treated both constructs as second-order factors to run structural equation modeling (SEM). The results showed acceptable model fit indices (*χ*^2^/*df* = 2.52, GFI = 0.93, AGFI = 0.91, CFI = 0.96, TLI = 0.95, SRMR = 0.05, RMSEA = 0.05) (Kline, 2005; Little, 2013). The findings showed that SAWBF are positively associated with organizational citizenship behavior [*β* = 0.76, SE = 0.06, CI_BC_ (0.72, 0.81)], and 58.4% of the variance in organizational citizenship behavior was explained. These results demonstrated the predictive validity correlations of SAWBF in relation to the oft-supported well-being outcome (i.e., organizational citizenship behavior).

**Figure 1:**
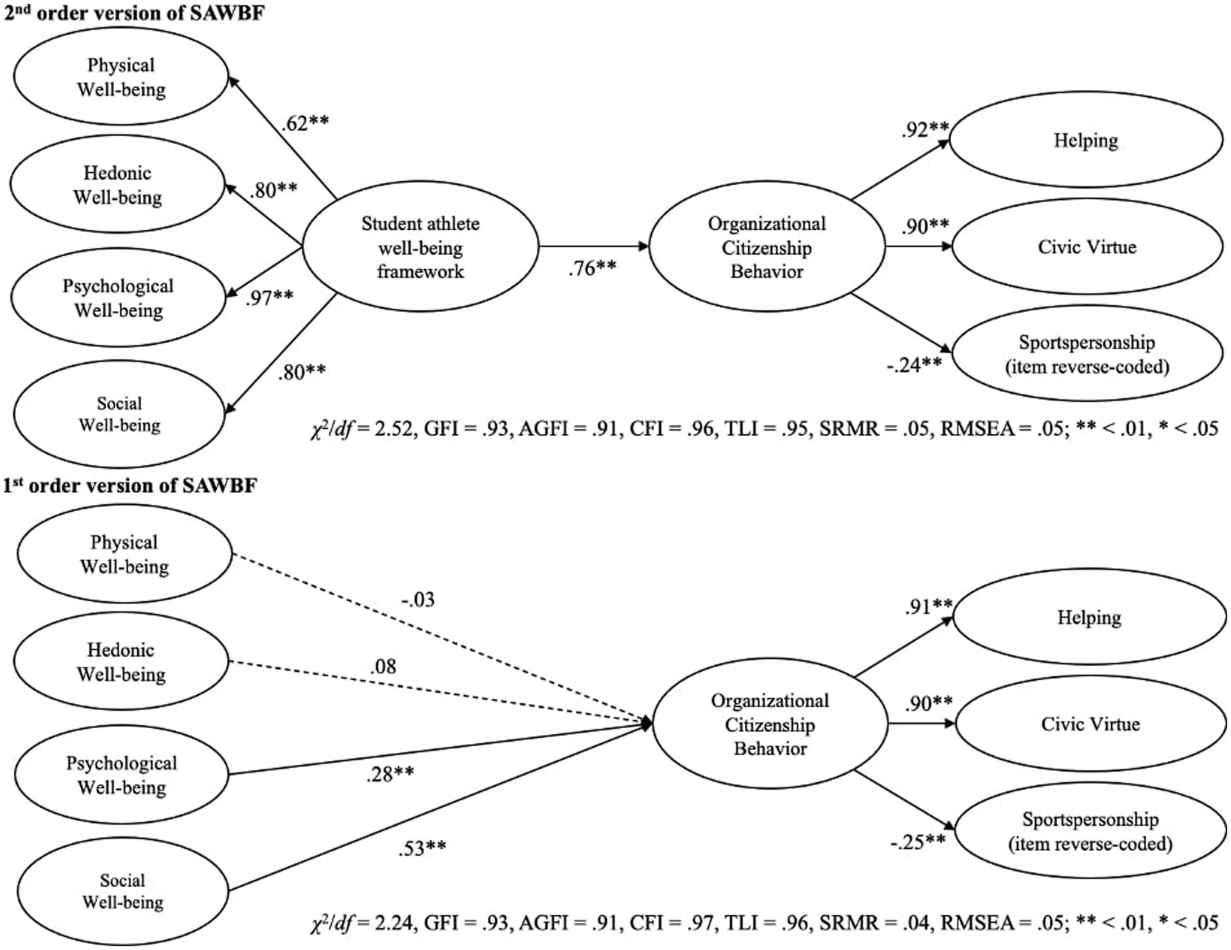
The results of structural equation modeling.

To further support the predictive validity correlations of SAWBF and understand the detailed associations with organizational citizenship behavior, we additionally ran SEM with the first-order SAWBF. The results showed acceptable model fit indices (*χ*^2^/*df* = 2.24, GFI = 0.93, AGFI = 0.91, CFI = 0.97, TLI = 0.96, SRMR = 0.04, RMSEA = 0.05; Kline, 2005; Little, 2013). The findings showed that physical and hedonic well-being were not associated with organizational citizenship behavior (*β* = −0.03, SE = 0.07, *p* = 0.58, CI_BC_ [−0.11, 0.06] for physical well-being; *β* = 0.08, SE = 0.06, *p* = 0.29, CI_BC_ [−0.07, 0.19] for hedonic well-being, respectively). Nevertheless, psychological well-being was found to be positively associated with organizational citizenship behavior [*β* = 0.28, SE = 0.11, *p* < 0.01, CI_BC_ (0.07, 0.43)]. Among SAWBF factors, social well-being was most strongly associated with organizational citizenship behavior [*β* = 0.53, *SE* = 0.13, *p* < 0.01, CI_BC_ (0.41, 0.65)]. Overall, 63.1% of the variance in organizational citizenship behavior was explained Figure 1 shows the visual summary of the structural equation models.

## 5. Discussion

### 5.1. Theoretical implications

The current study was aimed to validate the multidimensional (i.e., physical, hedonic, psychological, and social well-being) student athlete well-being framework–SAWBF. The authors also examined the relationship between SAWBF and organizational citizenship behavior to further demonstrate the predictive validity correlations of the framework. The data with 546 Japanese elite collegiate student athletes were procured to achieve the above purposes.

The findings of the current research can theoretically contribute to the literature in several ways. First, SAWBF is a theoretically comprehensive well-being framework. Previous literature has utilized various concepts related to well-being such as life satisfaction (Park et al., 2022), happiness (Matsumoto et al., 2018), and subjective well-being (Diener and Ryan, 2009). However, these concepts are highly related to the hedonic aspect of well-being only. Scholars have already acknowledged that physical conditions can give significant impact on athletes (Reardon et al., 2019). Moreover, considering student athletes’ environment where learning and growth are emphasized, it is important to incorporate eudaimonic aspects (i.e., psychological and social) of well-being (Kinoshita and Sato, 2023). Incorporating the above ideas, SAWBF, a multi-dimensional student athlete well-being framework including physical, hedonic, psychological, and social well-being aspects, is indeed theoretically useful. The current investigation successfully ensured its reliability and validity with the relatively succinct scale.

Second, the predictive validity correlations of SAWBF were also ensured. Various research has been conducted to explain potential antecedents of well-being (Lundqvist and Sandin, 2014; Lundqvist and Raglin, 2015; Kinoshita and Sato, 2023). While such scholarly efforts should be sustained, dependent variables that athletes prioritize may not always match with the ones of team managers and staff members (e.g., effective team management, winning). Hence, it is also imperative to highlight potential positive outcomes generated by well-being as it can be a driving force for team managers and staff members to take well-being of student athletes seriously. The current research demonstrated that SAWBF was associated with organizational citizenship behavior, one of the positive well-being outcomes (Figure 1). The findings are encouraging not only for well-being researchers, but also student athletes and their team managers/staff members as improving well-being of student athletes can eventually contribute to effective team management. To be further specific, the first-order model indicated that psychological and social well-being were particularly associated with organizational citizenship behavior, relative to physical and hedonic well-being. The results were consistent with the previous literature, demonstrating the positive associations between well-being and organizational citizenship behavior in various research contexts (e.g., Aoyagi et al., 2008; Paul et al., 2019; Yu et al., 2021). In this sense, the current research provided additional insights regarding the outcome of well-being focusing on the contexts of student-athletes.

### 5.2. Practical implication for athletes, coaches, and managers

Student athletes are exposed to various stressors (e.g., injury, performances, social pressure, fatigue; Rice et al., 2016). The expectations toward student athletes to well perform a dual role as athletes and students have kept rising (Van Rensburg et al., 2011). Given the situation, it is essential for practitioners (e.g., coaches and managers) to multi-dimensionally understand well-being of student athletes so that they can effectively support their athletes. From the practical standpoint, the supported relationship between SAWBF and organizational citizenship behavior can provide meaningful practical value of well-being.

The findings indicated that student athletes in Japan who have a sense of learning, confidence, and satisfying relationships with team members are more likely to contribute to the effective management of the team. In this sense, team managers and staff members can facilitate psychological and social well-being stimulating sport activities. For example, UNIVAS has launched the UNIVAS Student Lounge in 2021 where student athletes from member schools develop a marketing team to promote their games. Such activities outside of the fields can also provide learning experiences to student athletes, which may contribute to both psychological and social well-being. Although the findings emphasized the importance of psychological and social well-being in SAWBF, it does not mean the values of physical and hedonic well-being can be underestimated. The current research happened to find that organizational citizenship behavior was significantly associated with psychological and social well-being, but physical and hedonic well-being may be more strongly associated with other outcomes (e.g., low injury rate, sport continuation). Hence, athlete themselves and practitioners should pay attention to their well-being multi-dimensionally is an imperative task for sustainable growth.

The current research validated SAWBF based on elite collegiate student athletes in Japan. Yet, we employed cross-sectional design to understand the current state of students’ well-being. It is important to keep conducting student athlete well-being research to further deepen the understandings of athlete well-being. In fact, NCAA, which has often been referenced as a model case for UNIVAS, has conducted student-athlete well-being survey from 2020. Some may be surprised by the fact that such an influential sport organization has just recently started to understand their student-athletes’ well-being. Nevertheless, the efforts should be commended, and UNIVAS can also start investing the resources for understanding student-athletes’ well-being. Overall, the current research indicated the usefulness of SAWBF from both theoretical and practical perspectives.

## 6. Limitation and future research directions

There were several research limitations in this study. First, we employed a convenience sampling method to procure data through the authors’ research network. Although the samples were consistent with the target population, such a method is still limited in its ability to generalize the findings. Our data were obtained from student athletes in Kanto region (e.g., Tokyo, Kanagawa, Saitama), which may be considerably different from those in Kansai region (e.g., Osaka, Kyoto, Hyogo). Since the same concern is applicable from the cross-cultural perspectives, it is imperative to further conduct research to understand student athlete well-being based on various characteristics (e.g., region, culture, race). Related to above, the samples for the current research came from 23 different sport codes. While it could be beneficial in generalizability, it can also vanish the unique characteristics of each sport. Therefore, future research should be conducted to explore sport-based well-being among student-athletes.

Second, the findings regarding the outcomes of SAWBF are limited only to organizational citizenship behavior. It has been mentioned that well-being has the potential to give positive impact on health, work productivity, and resilience (Kansky, 2017). Related to this limitation, it should also be noted that the authors procured the data by a cross-sectional design, which does not provide robust causal implications. Hence, future research that incorporates SAWBF can also examine potential relationships with other adaptive outcomes by employing a longitudinal design.

Lastly, although students are often considered homogenous, there should be various moderators that can influence the relationship between SAWBF and organizational citizenship behavior. For example, gratitude may further enhance the impact of well-being on adaptive outcomes (Schnitker and Richardson, 2019). Similarly, mental toughness may serve as a shield to block the ill-being on maladaptive outcomes (Kinoshita et al., 2021). Future research therefore should be conducted to further enrich our understandings of well-being and its outcomes to make the sport environment better for student athletes.

## Data availability statement

The raw data supporting the conclusions of this article will be made available by the authors, without undue reservation.

## Ethics statement

The studies involving human participants were reviewed and approved by Waseda University. The patients/participants provided their written informed consent to participate in this study.

## Author contributions

SS and KK: conceptualization, data collection, data analyzes, and original draft writing. MK, YuY, and YaY: conceptualization and manuscript editing. HT: reviewing, editing manuscript, and supervision. All authors contributed to the article and approved the submitted version.

## Funding

The research was carried out as a part of research project funded by Grant-in-Aid for Early-Career Scientists of Japan Society for the Promotion of Science (JSPS KAKENHI grant number JP20292674).

## Conflict of interest

The authors declare that the research was conducted in the absence of any commercial or financial relationships that could be construed as a potential conflict of interest.

## Publisher’s note

All claims expressed in this article are solely those of the authors and do not necessarily represent those of their affiliated organizations, or those of the publisher, the editors and the reviewers. Any product that may be evaluated in this article, or claim that may be made by its manufacturer, is not guaranteed or endorsed by the publisher.

## Supplementary material

The Supplementary material (Japanese version of the questionnaire items) for this article can be found online at: https://www.frontiersin.org/articles/10.3389/fpsyg.2023.1171309/full#supplementary-material
