# Supplementary figures and images for: Student athlete well-being framework: an empirical examination of elite college student athletes
Source: Front Psychol. 2023 Jun 15;14:1171309. doi: 10.3389/fpsyg.2023.1171309 (PMC10313406; doi:10.3389/fpsyg.2023.1171309)

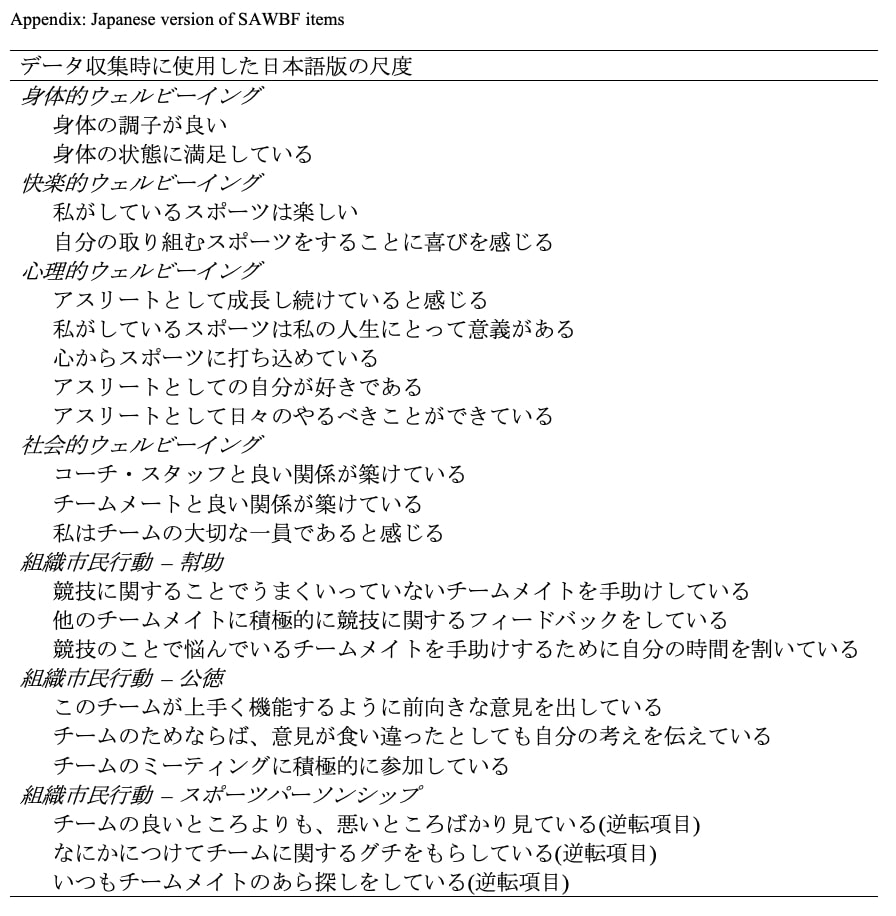

Supplement: Supplementary file 1 [file Image_1.JPEG]
